# Photocatalytic behavior for removal of methylene blue from aqueous solutions via nanocomposites based on Gd_2_O_3_/CdS and cellulose acetate nanofibers

**DOI:** 10.1007/s11356-023-28999-4

**Published:** 2023-08-24

**Authors:** Dalia Abdrabou, Mohamed Ahmed, Ali Hussein, Tharwat El-Sherbini

**Affiliations:** 1 Misr University for Science and Technology, 6 October, Giza, 12566 Egypt; 2 Department of Physics, Faculty of Science, Suez University, Suez, 43518 Egypt; 3 Laboratory of Laser and New Materials, Department of Physics, Faculty of Science, Cairo University, Giza, 12613 Egypt

**Keywords:** CdS, Gd_2_O_3_, Nanofiber, Wastewater, Methylene blue

## Abstract

Efficient cleaning of contaminated water by photocatalysis has become an effective strategy in recent years due to its environmental and ecological designation. Cadmium sulfate (CdS) is an excellent photocatalyst in the visible region but has low quantum efficiency. In order to increase the photocatalytic efficiency, CdS was modified with gadolinium oxide (Gd_2_O_3_) and combined with graphene oxide (GO) nanoparticles. The estimated crystallite size (D_s_) for Gd_2_O_3_, CdS/Gd_2_O_3_, and CdS/Gd_2_O_3_@GO was 29.6, 11.6, and 11.5 nm, respectively. The degradation of methylene blue (MB) reaches the highest values after 60 min under visible light irradiation with a dye concentration of (0.25 ppm). Whereas in powdered composition the efficiency of dye removal has been enhanced under UV irradiation, it reduced by increasing the MB concentration to 0.50 ppm with visible light irradiation. In addition, the CdS with/without Gd_2_O_3_ and GO were integrated into electrospun nanofibrous cellulose acetate (CA) through the electrospinning technique. The compounds of Gd_2_O_3_, CdS/Gd_2_O_3_, and CdS/Gd_2_O_3_/GO were encapsulated into CA nanofibers for the degradation of MB under visible and UV irradiation. The apparent rate constant (*k*) achieves a value of 0.006, 0.007, and 0.0013 min^−1^ while the removal efficiency reaches 41.02%, 54.71%, and 71.42% for Gd_2_O_3_@CA, CdS/Gd_2_O_3_@CA, and CdS/Gd_2_O_3_/GO@CA, respectively, after 60 min under UV irradiation.

## Introduction

Water pollution is considered one of the major problems that human beings face in the current decades (Chen et al. 2019; Moradnia et al. 2021). It includes different types of pollutants such as organic and inorganic ones (Gusain et al. 2019; Fang and Shangguan 2019; Yachao et al. 2020). Organic dyes have various types such as methylene orange (MO), rhodamine (B), and methylene blue (MB) (Vallejo et al. 2019; Liu et al. 2019). These dyes influence public health and might cause health issues to a lot of human beings (Gusain et al. 2019; Xiong et al. 2018; Sheik Mydeen et al. 2020; Kurniawan et al. 2023). In this regard, traditional methods involving physical adsorption, chemical precipitation, coagulation, flocculation, and photocatalysis have been used to eliminate these organic pollutants from aqueous solutions (Li et al. 2022; Alamgir et al. 2021). These procedures are nondestructive and transport organic contaminants from one state to another (Avilés-García et al. 2018). Among these technologies, photocatalysis is increasingly regarded as an appropriate choice in the last years (Qutub et al. 2022). Heterogeneous photocatalysis manifests as a useful alternative for the treatment of organic contaminants due to its green characteristic (Marques et al. 2021). It has many advantages such as simple process, economical, low energy exhaustion, eco-friendly, and high degradation activity for contaminant removal (Ponnamma et al. 2022; Yan et al. 2020). Numerous parameters affect the photocatalysis activity, such as size, shape, intensity, surface area, pH, and the amount of catalyst (Xu et al. 2021a). Among these, various semiconductors such as CdS, ZnO, TiO_2_, SnO_2_, and ZrO_2_ have been characterized as photocatalysts (Avilés-García et al. 2018). CdS is one of the utmost vastly survived semiconductor materials because of its bandgap (Qutub et al. 2022) and has been considered as an n-type (Li et al. 2020b). It is a superior photocatalyst in the visible light region but has weak stability and weak quantum efficiency (Rajendran et al. 2019). Furthermore, the photocatalytic activity of CdS is inferior due to photocorrosion and rapid recombination of photo-generated charge carriers (Aragon et al. 2019). The surface sulfide is oxidized to sulfur by photogenerated holes under radiation (Iqbal et al. 2019). Considering that photocatalytic interactions occur on the CdS surface, vital alterations are needed to stabilize the sulfide ions and transmit the photogenerated holes from the surface to prevent photocorrosion (Iqbal et al. 2019). This study aims to enhance the photocatalytic activity by depositing CdS to the sheets of graphene to acquire a uniform extended CdS and its conjugation with another semiconductor (Qutub et al. 2022). The materials’ components can be represented as electron carriers and disperse photogenerated electron–hole pairs to enhance efficiency (Xiang et al. 2019). The photocatalytic CdS compound is not only composed in a simple method, but can be utilized under UV and visible light states. The photocatalytic activity approaches approximately 91% degradation in 60 min exposure to our CdS powdered composition under UV light which is higher than pristine CdS in a previous work (Nagamine et al. 2020). Also, CdS–ZnO composite has the ability of improving their chemical and physical properties in order to increase the photocatalytic efficiency (Adegoke et al. 2019). The band energy structures of ZnO and CdS are suitable to stimulate the transfer of electron processes where photogenerated electrons can drift from CdS to ZnO (Ye et al. 2020). CdS/TiO_2_ photocatalytic material is composed via a simple process to develop the photocatalytic activity of TiO_2_ by sensitization of CdS. The degradation rate of CdS/TiO_2_ photocatalysts for MO was estimated under visible and UV irradiation states. The degradation efficiency of CdS/TiO_2_ photocatalyst under visible light was found to be lower than that under UV irradiation. Ertis and Boz (2017) surveyed the degradation of methylene blue (MB) using CdS nanoparticles doped with Ni, Co, Sb, and Ce. They detected that the efficiency of Co-doped CdS is higher than pristine CdS. The degradation efficiency was 87% of co-doped CdS after 4-h radiation time. Graphene oxide (GO) is a two-dimensional (2D) material (Alhaddad et al. 2021). It has attracted a massive deal of attention in critical applications due to several physical and chemical properties besides excellent electrical conductivity with a very high electron transfer speed of 2×10^5^ cm^2^V^−1^ s^−1^ (Mishra and Acharya 2020), (Singh et al. 2020). It might improve the photocatalytic activity to aggregate, develop charge carrier separation, and raise visible light absorption (Xu et al. 2021b). GO is a zero bandgap material (Alhaddad et al. 2021). It has been proven that graphene oxide disordered with nanoparticles such as CdS, ZnO, TiO_2_, and SnO_2_ and boosts photocatalytic reduction (Maruthupandy et al. 2020). Due to the possible CdS ion leaching, attempts to improve the photocatalytic efficiency of CdS have included changing the surface structure of CdS NPs by controlling morphology by depositing CdS to graphene sheets (Qutub et al. 2022). Moreover, highly water-soluble ultrathin and fluorescent CdS nanorods are directly synthesized with the assistance of polyethylenimine (PEI), a stable cationic polymer (Yue et al. 2022). On the other hand, gadolinium oxide is an n-type semiconductor and shows a magnetic property that can play a role to collect the adsorbents after the water cleaning process via an external magnetic field (Saravanan et al. 2018; Lingamdinne et al. 2021). Thus, the combination of gadolinium oxide and GO with CdS might improve the adsorption, separation, efficiency, and reusability of photocatalysts (Choi et al. 2020). Many researchers are exceedingly using functionalized GO-based magnetic materials for the possibility adsorption processing of wastewater polluted with heavy metals and organic materials (Lingamdinne et al. 2019). The CdS, Gd_2_O_3_, and GO powdered and nanofiber are being researched to find new material to enhance the catalyst efficiency to improve photocatalysis. The state of increasing demands in the field of semiconductor photocatalysis to boost new materials has motivated researchers toward progress. In the present work, we report the improved photocatalytic of CdS, Gd_2_O_3_, and GO composites powdered and nanofiber under visible and UV light. Besides that, cellulose acetate (CA) is a type of common semi-synthetic polymer obtained from cellulose (Chauhan et al. 2022). It possesses a lot of properties that are eligible for modifying semiconductors such as large surface area for interaction with water, water insolubility, high water absorption, retention capacity, good blinding nature, and remarkable ability to prevent phase separation (Na et al. 2021). The polymers can donate a suitable electronic space to limit the continuous growth of crystal grains and prevent aggregation (Rao and Ravikumar 2020). Moreover, the easy synthesis, high stability, and worthy environment compatibility of polymers are beneficial for functional application (Kolivand and Sharifnia 2020). Electrospinning is an efficient, less expensive, and the simplest method that uses a high electric field to produce nanofibers of polymeric solutions with diameters ranging from microns to nanometers (Liao et al. 2022). The electrospun nanomaterials represent numerous benefits, involving high porosity, high surface-to-volume ratio, functional features, and high flexibility (Chabalala et al. 2021; Huang et al. 2022). Thus, electrospinning applications have been used in various fields such as water purification, wound dressings, biosensors, tissue engineering, heterogeneous catalysis, and electronic devices (Mahmoud and Abdulhamid 2023). According to the reports of literature, few works have been classified to the improvement of CdS/Gd_2_O_3_/GO composition via the electrospinning process and minimally studied their photocatalytic creations in the degradation of the dye (Pascariu et al. 2022).

The present work aims to synthesize suitable powder and nanofiber composites using Gd_2_O_3_ and GO and CdS to develop photocatalytic activity, stability, and the ability to remove MB dyes, and to investigate the morphological, structural, and roughness features besides adsorption-desorption characteristic.

## Experimental details

### Materials

Cadmium chloride (CdCl_2_·2H_2_O; purity 98%), sodium sulfide (Na_2_S; 99.5%), and gadolinium oxide (Gd_2_O_3_; 99.0%) were purchased from LOBA (India). Graphene oxide (GO) (99.5%) was purchased from Sigma Aldrich and ammonium solution (25%), cellulose acetate (CA), and deionized water were used to prepare the solutions.

### Preparation of powder compositions

**Fig. 1:**
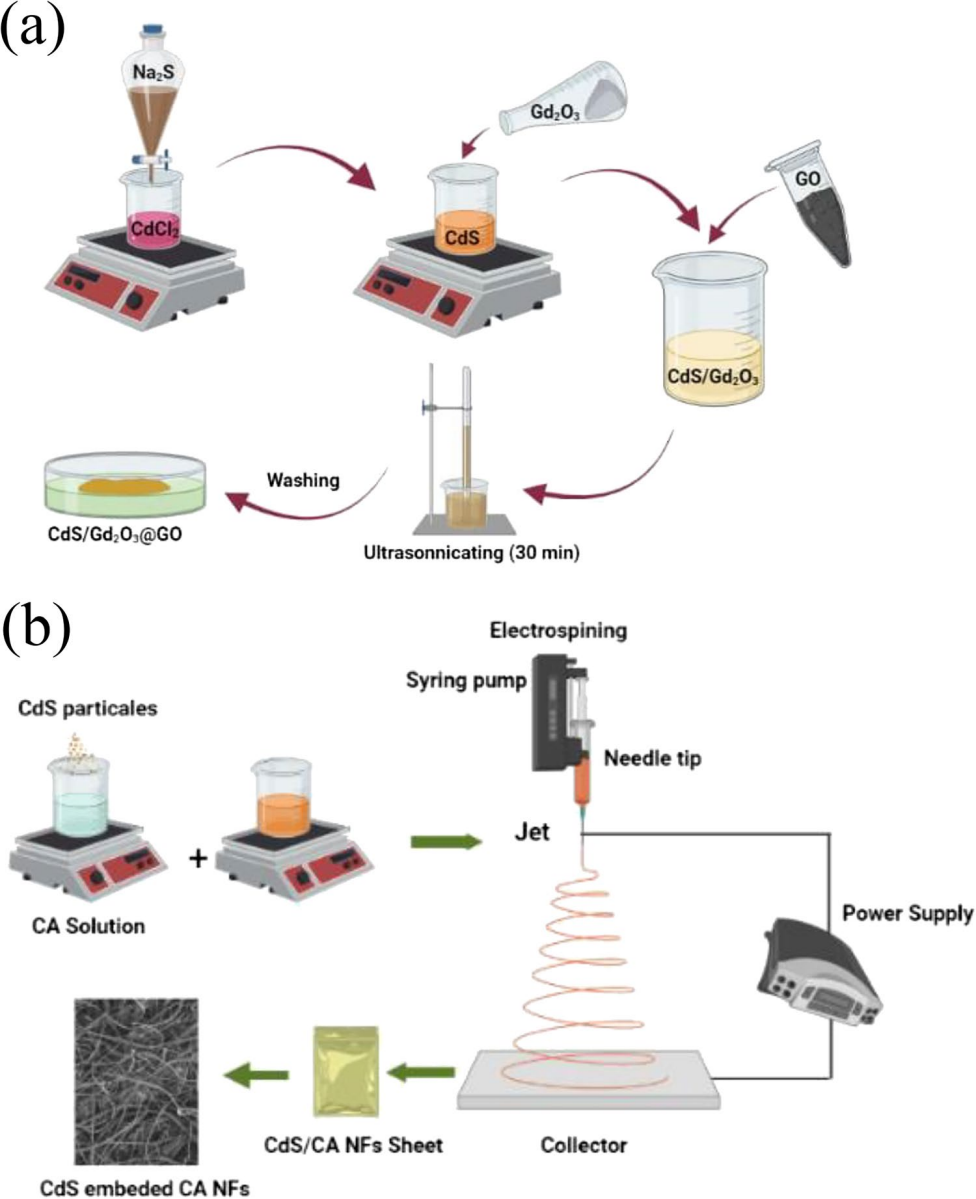
**a**, **b** Preparation of the powder and the nanofiber composition of CdS, CdS/Gd_2_O_3_, and CdS/Gd_2_O_3_@GO

The CdS was prepared using the co-precipitation method. CdCl_2_·2H_2_O (0.25 mol) and Na_2_S (0.09 mol) were dissolved in 50 mL of deionized (DI) water, separately. The CdCl_2_·2H_2_O solution was then dropped wisely into the Na_2_S container, while the pH value was kept at 10 using drops of NaOH. After 1 h of stirring, the solution was maintained for 24 h for precipitation. Equal weights of both gadolinium oxide (Gd_2_O_3_) and cadmium sulfide (CdS) were combined with and mixed in 50 mL of DI water under highly powerful sonication for 15 min. The solution was then left for precipitation and drying (CdS/Gd_2_O_3_). Also, 0.05 g of graphene oxide (GO) was combined with cadmium sulfide (CdS) and gadolinium oxide (Gd_2_O_3_) then sonicated, precipitated, and dried to get the sample of CdS/Gd_2_O_3_@GO as shown in Fig. 1a.

### Electrospinning procedure

In order to fabricate the samples of Gd_2_O_3_@CA, CdS/Gd_2_O_3_@CA, and CdS/Gd_2_O_3_/GO@CA nanofibers, 0.165 g of the acquired powder was added into 15 mL of CA (10 wt.%) in a glass bottle then sonicated for 30 min using an ultrasonic probe (Branson Digital Sonifier) to improve the dispersibility of nanofiber materials. The prepared electrospinning solution was transferred to a 20-mL syringe and proceeded with the flowing rate of injection: 1 mL h^−1^ via a syringe pump. The applied voltage was kept at 18 ± 0.1 kV, the distance from the needle tip to a collector was set as 16 cm, and the syringe needle was 22 φ. The electrospinning experiments were carried out at room temperature. The electrospun Gd_2_O_3_@CA, CdS/Gd_2_O_3_@CA, and CdS/Gd_2_O_3_/GO@CA were raised and accurately peeled off from the aluminum foil (Fig. 1b).

### Characterization

The crystal structure was analyzed by the XRD technique: Cu, k_α1_ target, λ = 1.5404 Å. The morphology of the composition was identified using a field emission scanning electron microscope. The elemental depiction was examined via energy-dispersive X-ray (EDX). Thus, the particle size and distribution were investigated using a transmission electron microscope. The optical properties were studied using UV–visible spectroscopy. In addition, XPS has characterized the chemical composition which was collected on K-ALPHA. Fourier-transmission infrared was used to characterize the functional groups of the matter surface. The surface area of the powdered compounds was checked using the Brunauer–Emmett–Teller (BET) technique.

### Dye degradation experiment

Methylene blue (MB) was utilized as an example of an organic pollutant. The photocatalytic activity was analyzed under both UV and visible light irradiation. To investigate dye degradation induced by UV irradiation, experiments were performed in 30 mL containers with MB concentrations of 0.25 and 0.5 ppm and 120 mg of nanofiber composition. These very low concentrations are used to prevent the rate of degradation from decreasing with increasing dye concentration (Vuppala et al. 2012). Prior to illumination, the mixing solution was conserved in a dark place for 60 min to achieve adsorption/desorption equilibrium and the dye solution concentration (*C*_0_) was defined as an initial concentration. A UV lamp of 15 W was placed about 12 cm above the dye solution and illuminated the sample for 60 min. At every interval time, 3 mL of the dye solution was taken by syringe to be investigated using UV–Vis spectrophotometer. In the degradation of dye under visible light*,* the experiment was carried out by halogen lamp (from 400 up to 800 nm at a power ~ 500 W) in a locked box at room temperature via a container of 30 mL with MB (0.25 and 0.5 ppm) and 100 mg of powdered compounds. The solution was kept in a dark place for 60 min and the distance between the halogen lamp and the sample was 18 cm. Every 10 min, the MB solution was detected by a spectrophotometer. The degradation efficiency (*η* %) was calculated via the following equation (Ponnamma et al. 2022; Ullah et al. 2020):

1 $$\eta =\left[\frac{\left({C}_{0 }-C\right)}{{C}_{0}}\right]\times 100\mathrm{ \%}$$

*C*_0_ and *C* are the initial and final concentrations of MB dye.

## Results and discussion

### XRD analysis

**Fig. 2:**
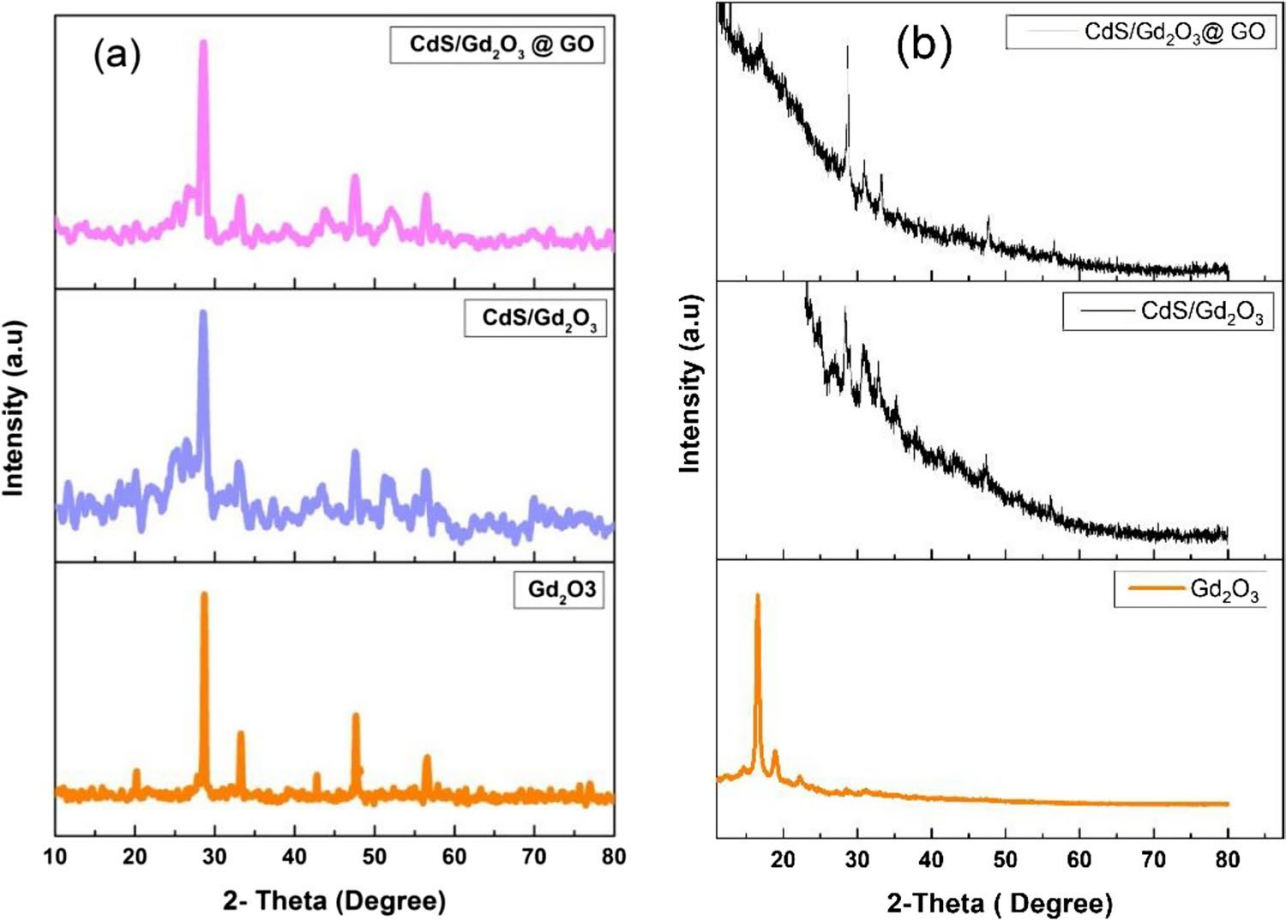
XRD pattern of pristine Gd_2_O_3_, CdS/Gd_2_O_3_, and CdS/Gd_2_O_3_@GO **a** powdered compositions and **b** nanofiber compositions

The diffraction peaks (Fig. 2a) analog to the Miller indices (211), (222), (400), (134), (440), (611), and (622) at reference card (00–043-1014) were corresponding to the cubic phase of Gd_2_O_3_, whereas the peaks (101), (110), (103), and (201) with reference card (00–006-0314) belong to the hexagonal system of CdS. The position of peaks seems to be with no significant shift, but the intensity decreased with CdS/Gd_2_O_3_. The crystallite size is calculated via Scherrer’s formula (Abdelmaksoud et al. 2021; Dixit et al. 2020):

2 $${D}_{\mathrm{s}}=\frac{K\lambda }{\beta \mathrm{cos}\theta }$$

where *D*_s_ is the crystalline size, *K* is a constant usually taken as 0.91, and *β* is the peak width at half-maximum height. However, the crystallite size of Scherrer does not take the lattice distortion into consideration; therefore, more accurate crystallite size according to Williamson–Hall (W–H) could be applied (Zhao et al. 2017). In this case,

3 $$\beta \mathrm{cos}\theta =\frac{K\lambda }{{D}_{\mathrm{s}}}+4\varepsilon \mathrm{sin}\theta$$

where *ɛ* is the lattice distortion.

Table 1 indicates that Scherrer’s crystallite size decreased from 29.6 to 11.5 nm for pure Gd_2_O_3_ and CdS/Gd_2_O_3_@GO, respectively. The negative sign of lattice distortion indicates the direction of the structural stress (Ahmed et al. 2020). The crystal size is calculated using the W–H equation, and it seems to be smaller than Sherrer’s one due to lattice distortion (Venkatesh et al. 2020). The XRD pattern of the Gd_2_O_3_@CA, CdS/Gd_2_O_3_@CA, and CdS/Gd_2_O_3_/GO@CA (Fig. 2b) displayed intense peaks, which manifested that these nanofibers have some degree of crystallinity. The main peaks of Gd_2_O_3_ and CdS are recognized with lower intensity than that in the powdered samples.

**Table 1:** Crystallographic data of Gd_2_O_3_, CdS/Gd_2_O_3_, and CdS/Gd_2_O_3_@GO powdered compositions including crystalline size (*D*_s_) via Scherrer’s equation, lattice distortion (*ε*), and uniform deformation model (UDM)

Composition	Sherrer’s crystallite size (nm)	ɛ×10^−2^	W–H crystallite size (nm)
Gd_2_O_3_	29.6	1.24	19.2
CdS/Gd_2_O_3_	11.6	− 2.87	8.3
CdS/Gd_2_O_3_/GO	11.5	1.09	8.2

### Morphology of powder and nanofibers

**Fig. 3:**
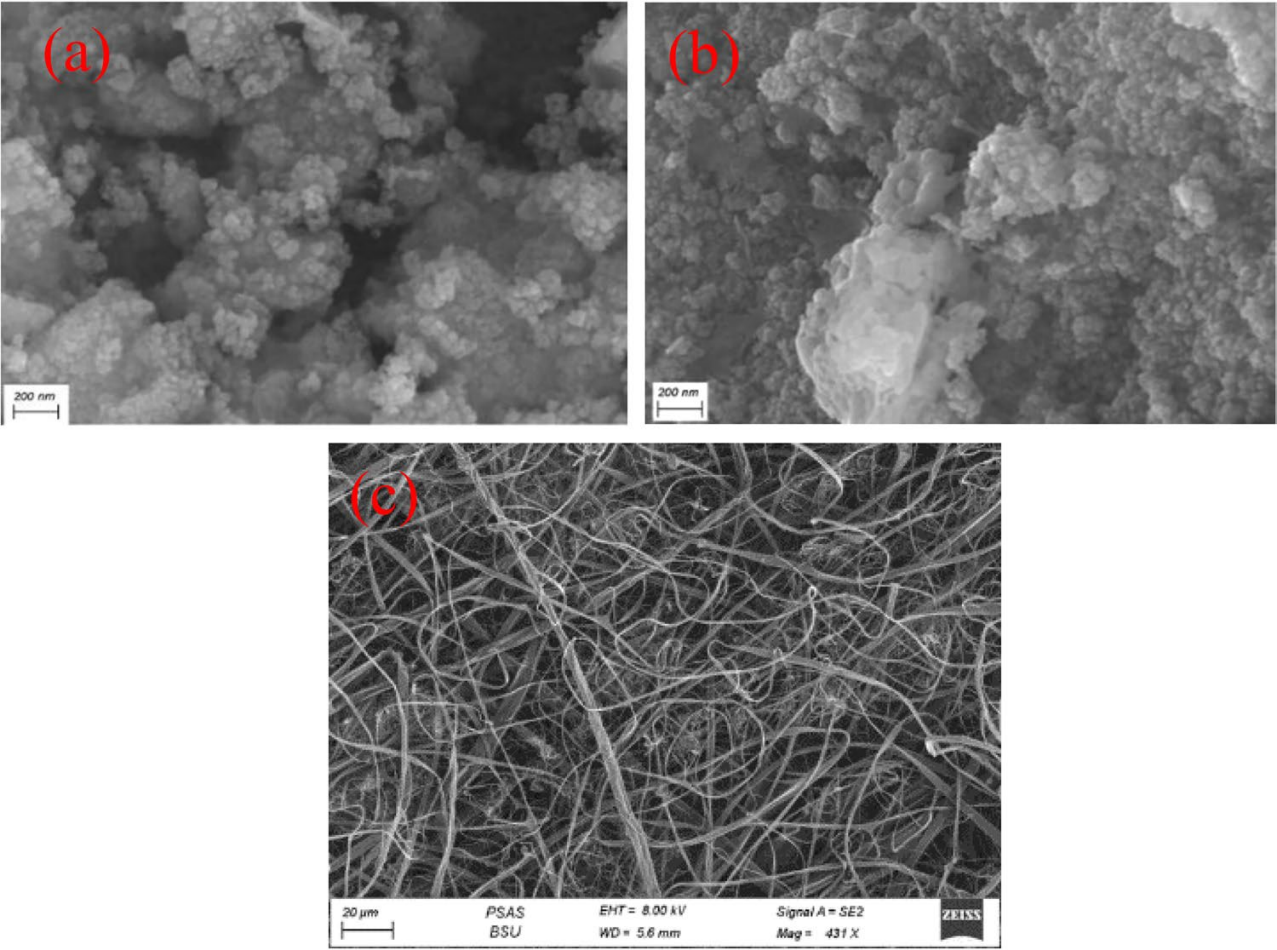
FE-SEM micrographs of the synthesized powdered composition of **a** CdS/Gd_2_O_3_, **b** CdS/Gd_2_O_3_@GO, **c** CdS/Gd_2_O_3_/GO@CA nanofiber

Scanning electron microscopy (SEM) was used to identify the morphology of the compounds. Although the images are not so clear due to some accidental problems, Fig. 3a shows that the CdS/Gd_2_O_3_ composite has a spherical form and a collection of irregular grain distribution. It elucidates a clear random aggregation and a rough structure. The composition has a grain size from 7.4 to 85.7 nm. Moreover, it contains cracks, internal porosity, and surface roughness. Figure 3b demonstrates that the particles of CdS/Gd_2_O_3_@GO are agglomerated randomly within the structure. The cracks and the surface roughness indicate the porousness of the prepared composites. The figure also displays high surface roughness besides the composition which has a flower-like structure. The grain size ranged from 11.1 to 66.9 nm. It indicates the structure of large clusters, low porosity, and disordered distribution. The high dispersibility of CdS particles within the surface of GO nanosheets might provide sufficient surface area, which is crucial for charge carrier generation (Ahmad et al. 2020). The high electrical conductivity of GO could increase the mobility of the generated electron, which can inhibit the recombination affinity. The morphology of the CdS/Gd_2_O_3_/GO@CA was investigated and is illustrated in Fig. 3c. The nanofibers seem to be formed in a cross-linked network with diameters around 25.7 nm up to 69.3 nm.

### EDX analysis

**Fig. 4:**
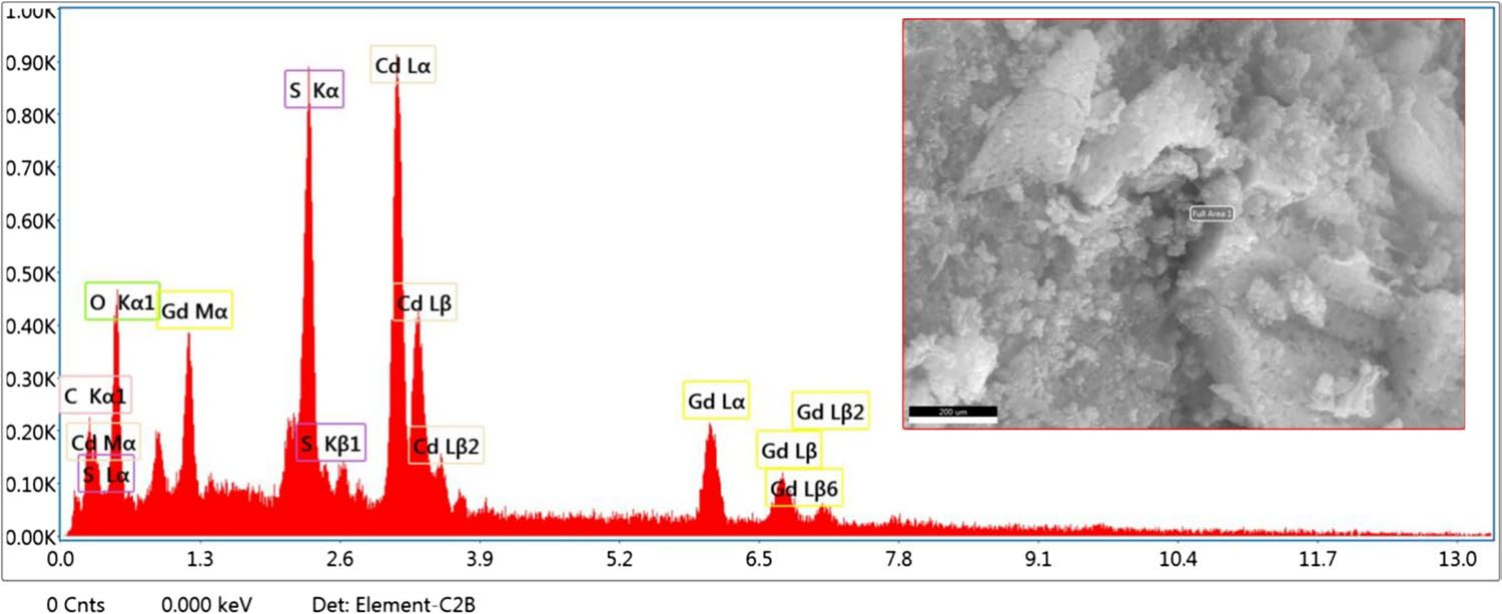
EDX analysis for CdS/Gd_2_O_3_@GO powdered compound

Elemental analysis was carried out by energy-dispersive X-ray (EDX) as shown in Fig. 4. The peaks coinciding to namely C (carbon), O (oxygen), S (sulfur), Cd (cadmium), and Gd (gadolinium) clearly appear and which show the characterized composition containing these traces and thus might confirm integration of the Gd_2_O_3_ and GO NPs with the CdS compounds. Furthermore, it confirmed the presence of C, S, Cd, and Gd amounts of CdS/Gd_2_O_3_@GO compound with 7.8, 16.7, 15.4, 35.9, and 24.0%, respectively. The atomic percentages are abbreviated in Table 2.

**Table 2:** EDX elemental analysis of the compounds

Element	Weight %	Atomic %
C K	7.8	24.7
O K	16.7	39.3
S K	15.4	18.1
Cd L	35.9	12.0
Gd L	24.0	5.7

### TEM studies for powdered and nanofiber compositions

**Fig. 5:**
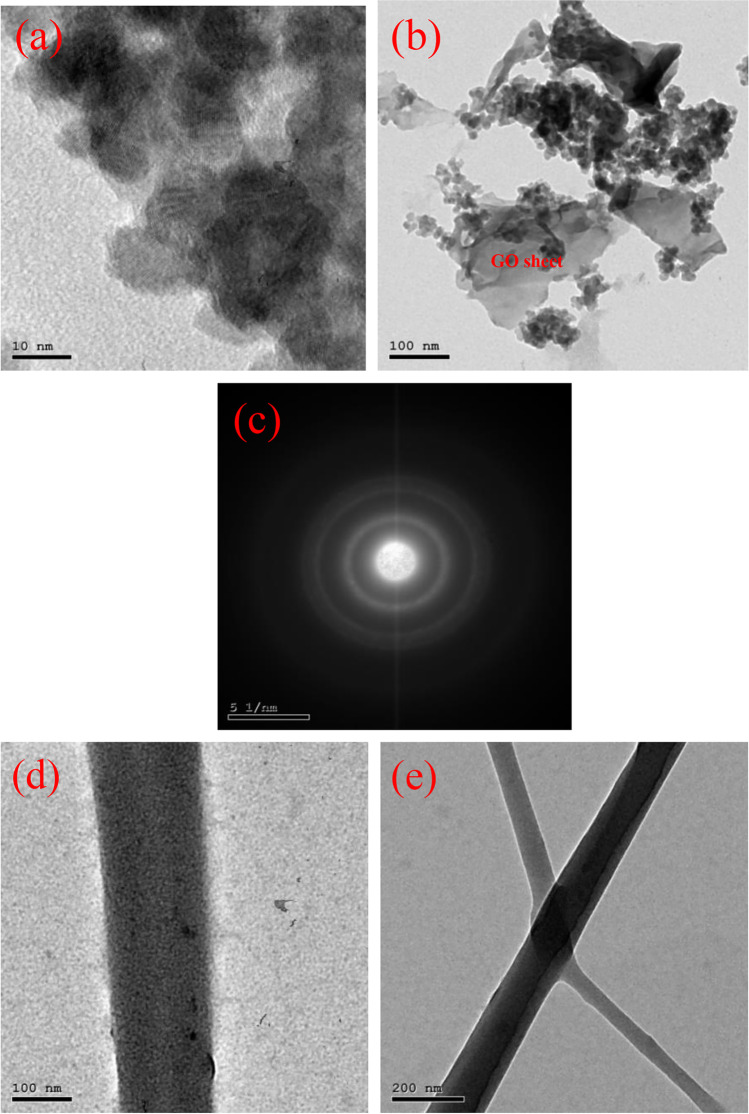
TEM micrographs of **a**–**c** CdS/Gd_2_O_3_@GO powder composition at several magnifications; **d**, **e** CdS/Gd_2_O_3_@GO nanofiber

The micrograph of CdS/Gd_2_O_3_/GO powdered is illustrated in Fig. 5. It could be demonstrated that GO have been formed in rectangular layers with a symmetrical distribution. The mixed composition exhibits irregular size distribution. On the other hand, the irregular shape and non-homogenous size distribution of CdS/Gd_2_O_3_ could be assigned to the cumulative influence of continual fragmentation (Ahmed and Imam 2020). The selected area electron diffraction (SAED) displays the crystalline network of CdS. Thus, SAED proves the polycrystalline nature of the composite.

Figure 5d and e shows that the CdS/Gd_2_O_3_/GO@CA nanofiber has a smooth surface. The spherical spots that seem to be incorporated in the nanofibers might be referred to the presence of CdS/Gd_2_O_3_/GO. CdS/Gd_2_O_3_/GO@CA was composed with diameters in the range of 10 nm.

### XPS studies

**Fig. 6:**
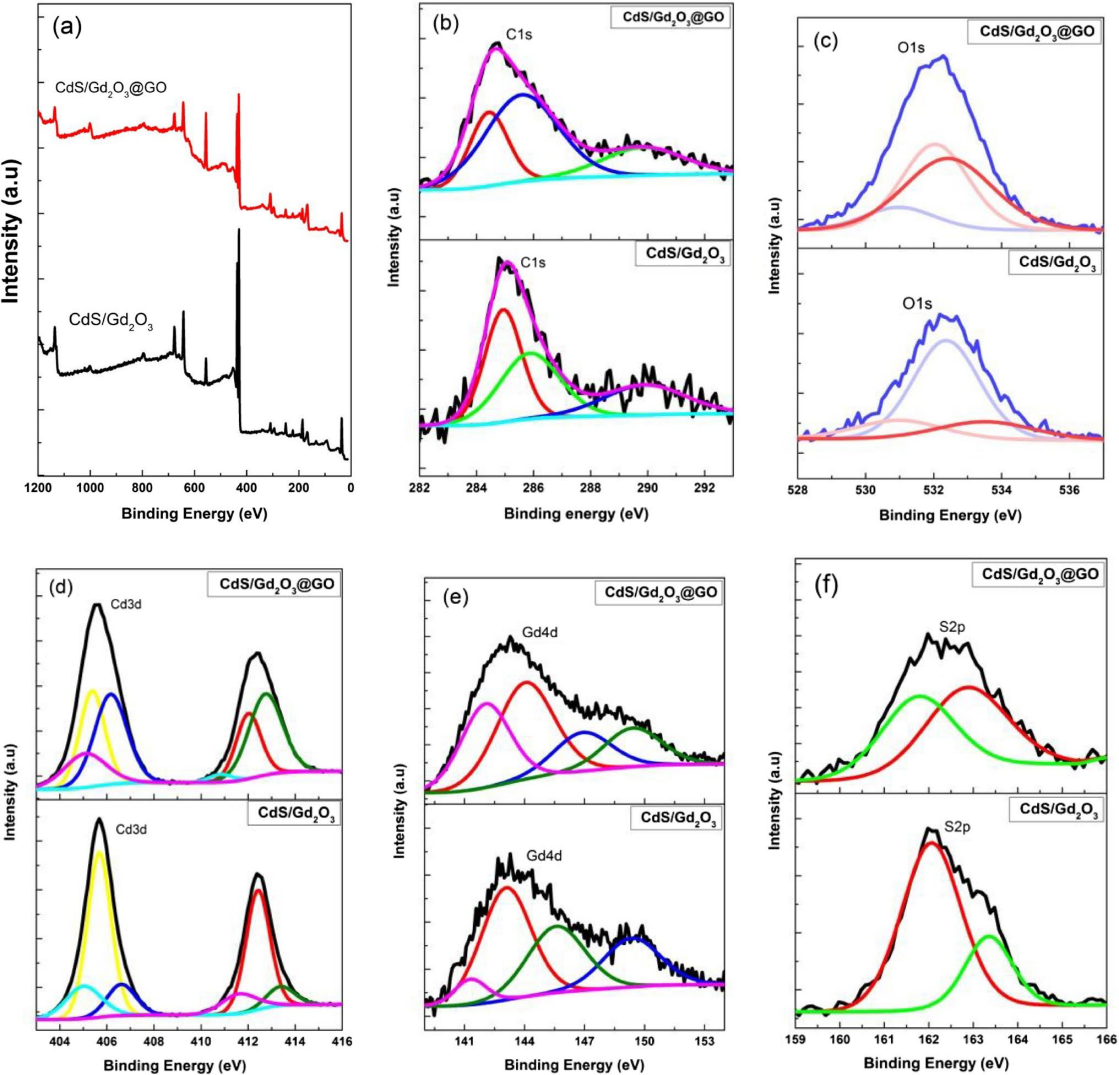
XPS spectra of CdS/Gd_2_O_3_ and CdS/Gd_2_O_3_@GO powdered compound: **a** survey, **b** C1s, **c** O1s, **d** Cd3d, **e** Gd4d, and **f** S2p

X-Ray photoelectron spectroscopy (XPS) is used to analyze the chemical composition and elements status of CdS/Gd_2_O_3_ and CdS/Gd_2_O_3_@GO (Selvaraj et al. 2019). The surface composition, the valence state of the elements, and functional group of CdS, Gd_2_O_3_, and GO were analyzed by XPS (Xing et al. 2020). The spectrum of XPS demonstrates that the composite is superimposed on Cd, C, O, Gd, and S. Figure 6a manifests the XPS spectra for the Cd, C, O, Gd, and S elements, which displays the existence of CdS, Gd_2_O_3_, and GO in the created composites. In Fig. 6b, the three deconvolution signals in the C1s spectrum were determined at 284.9, 285.8, and 289.8 eV and are referred to the C atoms of C–C/C = C, C–N, and N–C = N, respectively. As for Fig. 6c, the O1s peaks in the Gd_2_O_3_ and GO indicate the binding energy at Gd–O–Gd (230.9 e V), C = O (532.3 eV), Gd–O (531.3 eV), and OH group or a water molecule on the surface of nanocompositions (532.0 eV). The convoluted high-resolution spectrum elucidates that the Cd3d spectrum includes peaks at 412.3 eV (Cd3d3/2) and 405.6 eV (Cd3d5/2) which was symmetric with the particular binding energy of Cd^2+^ in CdS in Fig. 6d. In Fig. 6e, XPS spectrum of Gd4d region was executed to conclude the chemical state of Gd species on the surface of CdS/Gd_2_O_3_ and CdS/Gd_2_O_3_@GO photocatalyst. The peaks at 143.0 and 149.4 eV coincide with Gd4d5/2 and Gd4d3/2 transitions of Gd_2_O_3_. At the same time, the two single peaks of 163.3 eV (S2p1/2) and 161.7 eV (S2p3/2) estimated the particular peaks of the S^2−^ in the composition (Fig. 6f). The XPS analysis is demonstrated in Table 3.

**Table 3:** XPS spectra analysis for CdS/Gd_2_O_3_ and CdS/Gd_2_O_3_@GO

Composition	Band	Peak BE (eV)	Atomic %	Assignment	Ref
CdS/Gd_2_O_3_	C1s	284.9	39.5	C–C/C = C	(Liu et al. 2021)
	C1s scan A	285.8	37.2	C–N	(Liu et al. 2021)
	C1s scan B	289.8	23.2	π–π	(Li et al. 2021)
	O1s	532.3	65.6	C = O	(Liu et al. 2021)
	O1s scan A	530.9	16.8	Gd–O–Gd	(Lingamdinne et al. 2021)
	Cd3d	405.6	38.8	Cd 3d5/2	(Xing et al. 2020)
	Cd3d scan A	412.4	28.6	Cd 3d3/2	(Xing et al. 2020)
	Cd3d scan B	406.6	8.9	Cd 3d5/ 2	(Li et al. 2021)
	Cd3d scan C	413.3	5.2	Cd3d3/2	(Kande et al. 2019)
	Cd3d scan D	405.0	11.4	Cd 3d5/2	(Liu et al. 2021)
	Cd3d scan E	411.6	6.8	Cd 3d3/2	(Liu et al. 2021)
	Gd4d	143.0	42.9	Gd 4d5/2	(Barrera et al. 2018)
	Gd4d scan A	149.3	21.4	Gd 4d3/2	(Barrera et al. 2018)
	Gd4d scan B	145.5	29.2	Gd 4d3/2	(Sugyeong et al. 2021)
	Gd4d scan C	141.3	6.3	Gd 4d5/2	(Sugyeong et al. 2021)
	S2p	162.0	75.2	S 2p1/2	(Liu et al. 2021)
	S2p scan A	163.3	24.7	S 2p1/2	(Xing et al. 2020)
CdS/Gd_2_O_3_@GO	C1s	284.4	24.4	C–C/C = C	(Liu et al. 2021)
	C1s Scan A	289.8	20.0	π–π	(Li et al. 2021)
	C1s Scan B	285.5	55.5	C–N	(Liu et al. 2021)
	O1s	531.3	15.6	Gd–O	(Lingamdinne et al. 2021)
	O1s Scan C	532.0	38.4	OH group	(Kumaresan et al. 2020)
	O1s Scan E	532.2	45.8	C = O	(Liu et al. 2021)
	Cd3d	405.3	21.2	Cd 3d5/2	(Liu et al. 2021)
	Cd3d Scan A	412.0	14.3	Cd 3d3/2	(Xing et al. 2020)
	Cd3d Scan B	406.1	25.9	Cd 3d5/2	(Li et al. 2021)
	Cd3d Scan C	412.7	24.3	Cd 3d3/2	(Liu et al. 2021)
	Cd3d Scan D	410.8	1.6	Cd 3d3/2	(Xia et al. 2019)
	Cd3d Scan E	405.0	12.4	Cd 3d 3/2	(Yang et al. 2019)
	Gd4d	144.0	39.2	Gd 4d5/2	(Barrera et al. 2018)
	Gd4d Scan B	146.9	15.7	Gd 4d3/2	(Sugyeong et al. 2021)
	Gd4d Scan C	149.4	15.7	Gd 4d3/2	(Barrera et al. 2018)
	Gd4d Scan D	142.0	29.1	Gd 4d5/2	(Sugyeong et al. 2021)
	S2p	162.8	53.4	S 2p1/2	(Liu et al. 2021)
	S2p Scan A	161.7	46.6	S 2p3/2	(Liu et al. 2021)

### FT-IR analysis

**Fig. 7:**
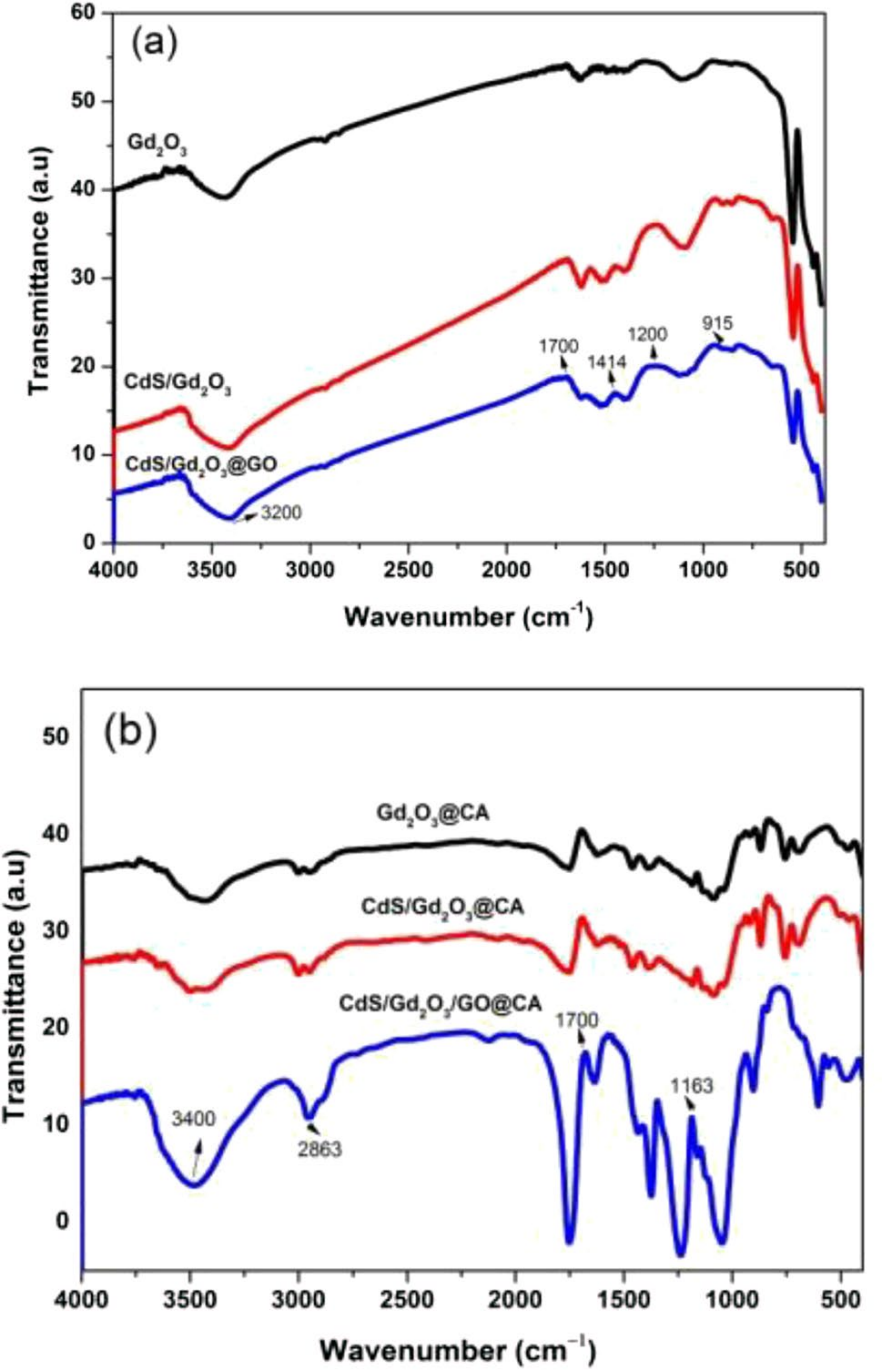
FT-IR spectra of transmittance **a** powdered composition, **b** nanofiber composition

The FT-IR spectra are shown in Fig. 7 where the main bands are reported in Table 4. As it was observed in Fig. 7a, the band at 540 cm^–1^ is ascribed to Gd–O stretching in Gd_2_O_3_. The spectra of GO that manifest a peak at 1042 cm^−1^ can be assigned to the C–O stretching. The broadband of 3271 cm^−1^ could be attributed to the stretching vibration mode of O–H. The stretching vibrations of hydroxyl (OH) groups of water adsorbed by the samples were ascribed to the broad peak shown at 3100–3600 cm^−1^. The peaks at 2921 and 2854 cm^−1^ indicate the existence of stretching vibration of C–H. The band of 1635 cm^−1^ is attributed to O–H–O bending oscillations because the molecules of water are adsorbed on the composite’s surface. The exposed band of 1594 cm^−1^ is assigned to the asymmetric vibrational mode belonging to the carboxyl group (C = O). The bands concerning 1414 and 1326 cm^−1^ manifest the existence of CH_2_ and C–O bending, respectively. The bands at 1048 to 843 cm^−1^ are referred to the stretching of C–O and the bending mode of O–H vibration, respectively. In Fig. 7b, the broadband of the 3200–3700 cm^−1^ in the spectra is attributed to the symmetric and anti-symmetric stretching of O–H vibrations. In addition, the band at 1625 cm^−1^ is concerned with the bending vibrations of absorbed water on the surface of nanofibers. The band 1633 cm^−1^ is assigned to the bending mode of H–O–H. The peak at 1102 to 1180 cm^−1^ is attributed to C–O–C stretching.

**Table 4:** The main characteristic bands of FT-IR

Gd_2_O_3_	CdS/Gd_2_O_3_	CdS/Gd_2_O_3_@GO	Gd_2_O_3_@CA	CdS/Gd_2_O_3_@CA	CdS/Gd_2_O_3_/GO@CA	Assignment	Ref
–	–	–	1419	1421	1670	C–H bending	(Qutub et al. 2022)
3430	3400	3420	3400	3470	–	O–H vibration	(Pascariu et al. 2022)
540	–	–	–	–	–	Gd–O stretching	(Christophe Massard 2022)
–	–	–	–	–	1570	O–H bond	(Liu et al. 2021)
1633	–	–	–	–	1633	H–O–H bending	(Ni et al. 2021)
–	–	–	–	–	1344	Cd–S bond	(Liu et al. 2021)
915	–	1042	843	1709	–	C–O stretching	(Lingamdinne et al. 2021)
–	–	–	1630	–	–	O–H–O bending	(Abu-Dief et al. 2020)
–	–	1102	1160	1159	1180	C–O–C stretching	(Liu et al. 2021)

### Surface area

**Fig. 8:**
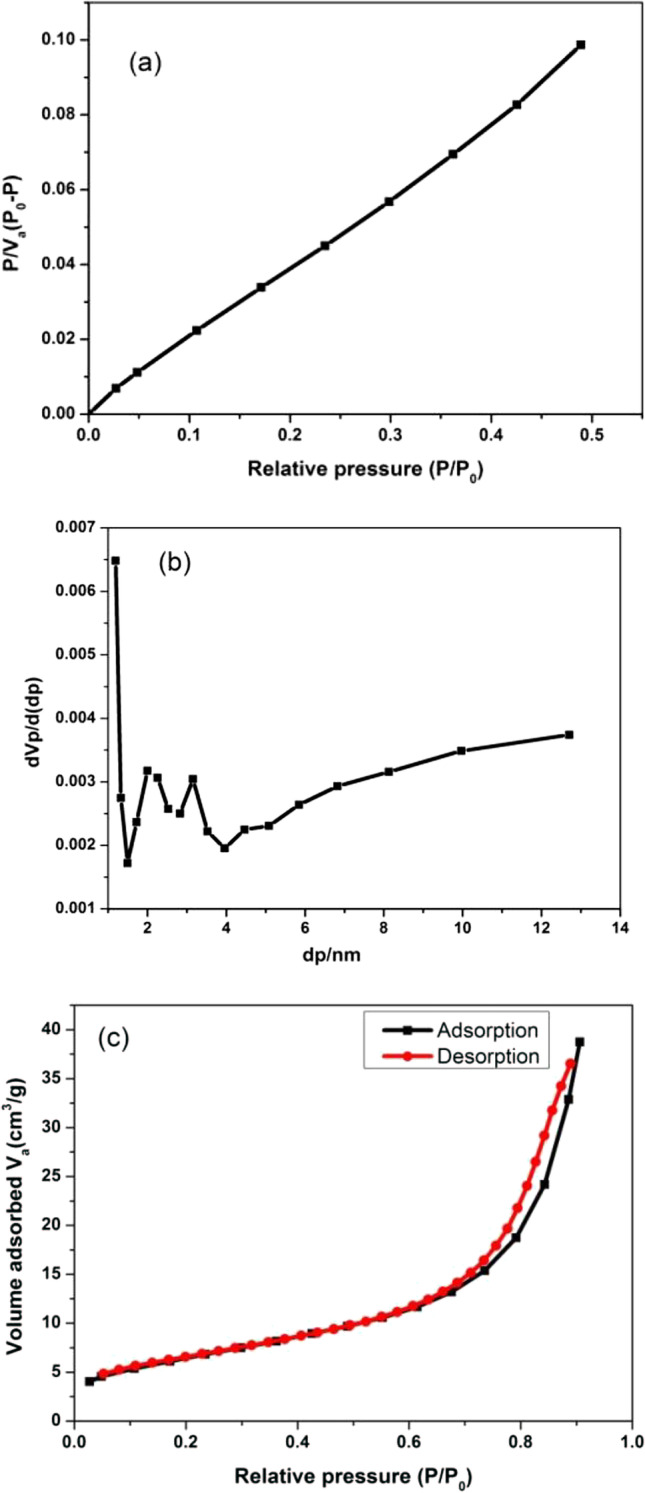
**a** BET surface area, **b** BJH pore size distribution, and **c** adsorption–desorption isotherms of N_2_ adsorption at 77.36 K for CdS/Gd_2_O_3_@GO powdered composition

The surface area and particle size are crucial for the adsorption ability while the pored structures are demanded in the field of wastewater treatment (Li et al. 2020a). The specific surface area and the pore size distribution of the powdered composition were investigated via nitrogen (N_2_) adsorption/desorption isotherms using the Brunauer–Emmett–Teller (BET) method at 77 K (Fig. 8). The existence of pores in broad size distribution could be ascribed to the intra- and inter-combination of monocular crystals through the formalization of powdered structure. The porosity of a matter is realized as the average of the pore volume divided by the total volume (AlAbduljabbar et al. 2021). The pore size distribution was resolved using the BJH (Barrett–Joyner–Halenda) procedure (Lee et al. 2021).

The surface area (*S*_BET_) was about 24 m^2^/g as demonstrated in Table 5. It was found that the total pore volume is 0.059 (cm^3^/g), whereas the average pore diameter reached 9.95 nm. The BET surface area of CdS loaded on activated carbon AC/CdS and pure CdS were 69.4 and 27.5 m^2^/g (Kande et al. 2019).

**Table 5:** Specific surface area (*S*_BET_), pore volume (*V*_p_), total pore volume, average pore diameter, and relative pressure (*P*/*P*_0_) of catalysts were determined from N_2_ adsorption at 77 K using the BET method

Composition	Surface area *S*_BET_ (m^2^/g)	Pore volume *V*_p_ (cm^3^/g)	Total pore volume (cm^3^/g)	Average pore diameter (*D*_p_) (nm)	Relative pressure (*P*/*P*_0_)
CdS/Gd_2_O_3_@GO	24	0.040	0.059	9.95	0.906

### Photocatalytic degradation experiment

#### Photocatalytic activity of powdered compositions under visible and UV light with concentration 0.25 ppm

**Fig. 9:**
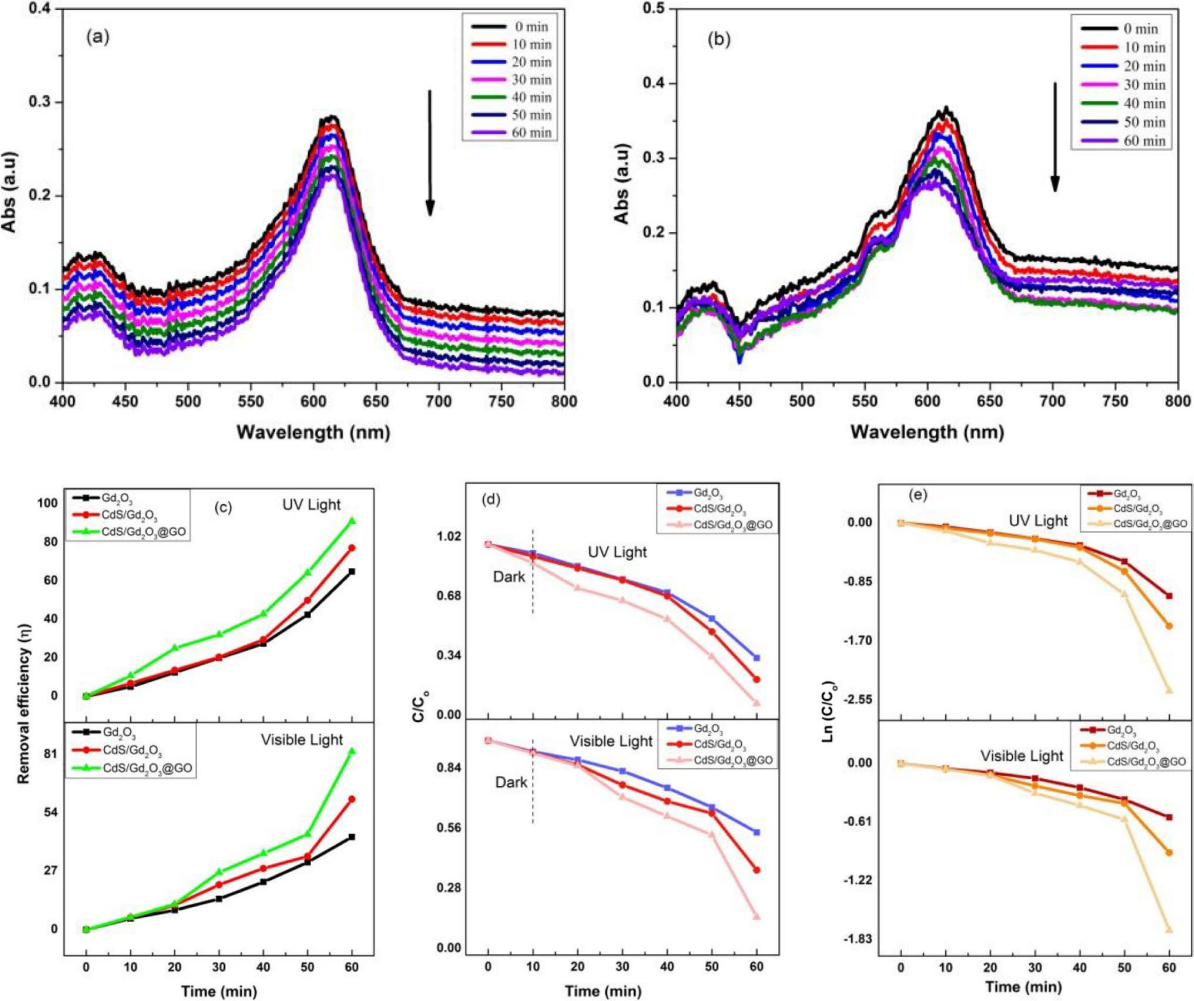
**a**, **b** Adsorption peaks of powdered CdS/Gd_2_O_3_@GO under visible and UV light with 0.25 ppm, **c** degradation efficiency with 0.25 ppm, **d** plot *C*/*C*_0_ and time with 0.25 ppm, **e** the identical pseudo-first-order kinetics of compounds for the degradation of MB with 0.25 ppm

The degradation of methylene blue is vastly used as an example to characterize the efficacy of the photocatalysts in the wastewater treatment process (Nasser et al. 2022). Figure 9a and b explicates the absorption spectra of the MB solutions with/without catalyst. For the MB solution with Gd_2_O_3_, CdS/Gd_2_O_3_, and CdS/Gd_2_O_3_@GO compositions, the distinctive peaks of MB at the major absorption wavelengths (664 nm) progressively decline slowly with increasing irradiation time. It is emphasized that embossing CdS with Gd_2_O_3_ and Gd_2_O_3_@GO develops photocatalytic activity in the visible and UV region. It is depicted in Fig. 9c that the light irradiation time was changed (up to 60 min). The degradation efficiency (*η*) reaches the highest values at 65%, 77%, and 91.07% for Gd_2_O_3_, CdS/Gd_2_O_3_, and CdS/Gd_2_O_3_@GO under UV light after (60 min) of irradiation time. However, the degradation efficiency under visible irradiation tends to be lower than its analogue under UV illumination. The efficiency (*η*) is ranged from 42.85%, 60.37%, and 82.35% for Gd_2_O_3_, CdS/Gd_2_O_3_, and CdS/Gd_2_O_3_@GO under visible light, respectively. As shown in Fig. 9d, the analysis of the photocatalytic properties, the reaction kinetics using the Langmuir–Hinshelwood (L–H) model for pseudo-first-order degradation rate is applied (Yao et al. 2021). Table 6 shows the values of the degradation rate constant *k*. Notably, it is well assured that the photocatalytic degradation of MB is obeyed as the first-order kinetics defined by the following equation (Huang et al. 2022):

4 $$\mathrm{Ln} \left[\frac{C}{{C}_{0}}\right]= - {k}_{\mathrm{app}}t$$

where *C*_0_ is the initial concentration of the MB at *t* = 0, *C* is the dye’s concentration at different interval times, and *k* is the reaction rate constant (Liao et al. 2022). Figure 6e exhibits the relationship between Ln (*C*_0_/*C*) and irradiation time which expands the correlation coefficient *R*^2^ and rate constant *k* for the MB dye degradation. The degradation ratio of MB is changed with the use of several photocatalysts under visible and UV irradiation as extended in Fig. 9d. The correlation coefficient *R*^2^ and *k* were calculated by plotting *t*/*qt* versus *t*, where *q*_*t*_ represents the amount of MB absorbed at time *t*. The maximum adsorption quantity (*q*_max_) was calculated to determine the conversion capacity value using the following equation (Abbas and Trari 2020):

5 $${q}_{\mathrm{max}}(\mathrm{mg}/\mathrm{g})=\frac{\left({C}_{0}-{C}_{\mathrm{e}}\right) V}{m}$$

where *q*_max_ acts as the optimal adsorbed quantity of MB, *C*_e_ is the final concentration (mg/L), *V* is the volume of working solution (L), and *m* is the mass of the catalyst. The pseudo-first-order kinetic model is used to estimate the interaction between MB molecules and catalysts. Table 6 gives the estimated values of the reaction rate constant *k*_app_ for all samples under visible and UV irradiation, while Table 7 lists the values of kinetics parameter *k*1, *q*_max_ according to L–H model, in visible and UV illumination for all samples, with *R*^2^ ranging between 0.877 and 0.999. The similarity between calculated *q*_max_ and those determined experimentally indicates that MB adsorption obeys pseudo-first-order kinetics for all adsorbents (Khalil Lazaar et al. 2021).

**Table 6:** The values for *k*_app_ (min^−1^) for powdered and nanofiber composites with a concentration of 0.25 ppm

Catalysts		Light source	*k*_app_ (min^−1^) × 10^−3^	*R*^2^
Powder	Gd_2_O_3_		8.8	0.999
	CdS/Gd_2_O_3_	Visible	1.32	0.906
	CdS/Gd_2_O_3_@GO	*(Halogen lamp, 500 W)*	2.3	0.877
	Gd_2_O_3_		1.55	0.999
	CdS/Gd_2_O_3_	UV	2.1	0.998
Nanofiber	CdS/Gd_2_O_3_@GO	*(UVC lamp, 15 W)*	3.3	0.997
	Gd_2_O_3_		6.0	0.998
	CdS/Gd_2_O_3_	Visible	7.0	0.998
	CdS/Gd_2_O_3_@GO	*(Halogen lamp, 500 W)*	1.3	0.985
	Gd_2_O_3_		7.0	0.999
	CdS/Gd_2_O_3_	UV	1.09	0.878
	CdS/Gd_2_O_3_@GO	*(UVC lamp, 15 W)*	1.8	0.905

**Table 7:** The kinetic parameters of different compositions for the Langmuir–Hinshewood (L–H) model under visible and UV irradiation with concentration 0.25 ppm

Catalysts		Light source	*k*1	*q*_max_ (mg/g)
Powder	Gd_2_O_3_		− 0.77	4.32
	CdS/Gd_2_O_3_	Visible	− 4.92	− 144.92
	CdS/Gd_2_O_3_@GO	*(Halogen lamp, 500 W)*	− 6.58	− 126.58
Powder	Gd_2_O_3_		− 0.4	8.33
	CdS/Gd_2_O_3_	UV	− 0.43	7.69
	CdS/Gd_2_O_3_@GO	*(UVC Lamp, 15 W)*	− 0.26	12.5
Nanofiber	Gd_2_O_3_		− 0.66	5
	CdS/Gd_2_O_3_	Visible	− 0.59	5.64
	CdS/Gd_2_O_3_@GO	*(Halogen Lamp, 500 W)*	− 0.38	8.69
	Gd_2_O_3_		− 0.76	4.34
	CdS/Gd_2_O_3_	UV	− 0.87	3.83
	CdS/Gd_2_O_3_@GO	*(UVC Lamp, 15 W)*	− 0.4	8.33

#### Photocatalytic activity of powdered compositions under visible light with concentration 0.5 ppm

**Fig. 10:**
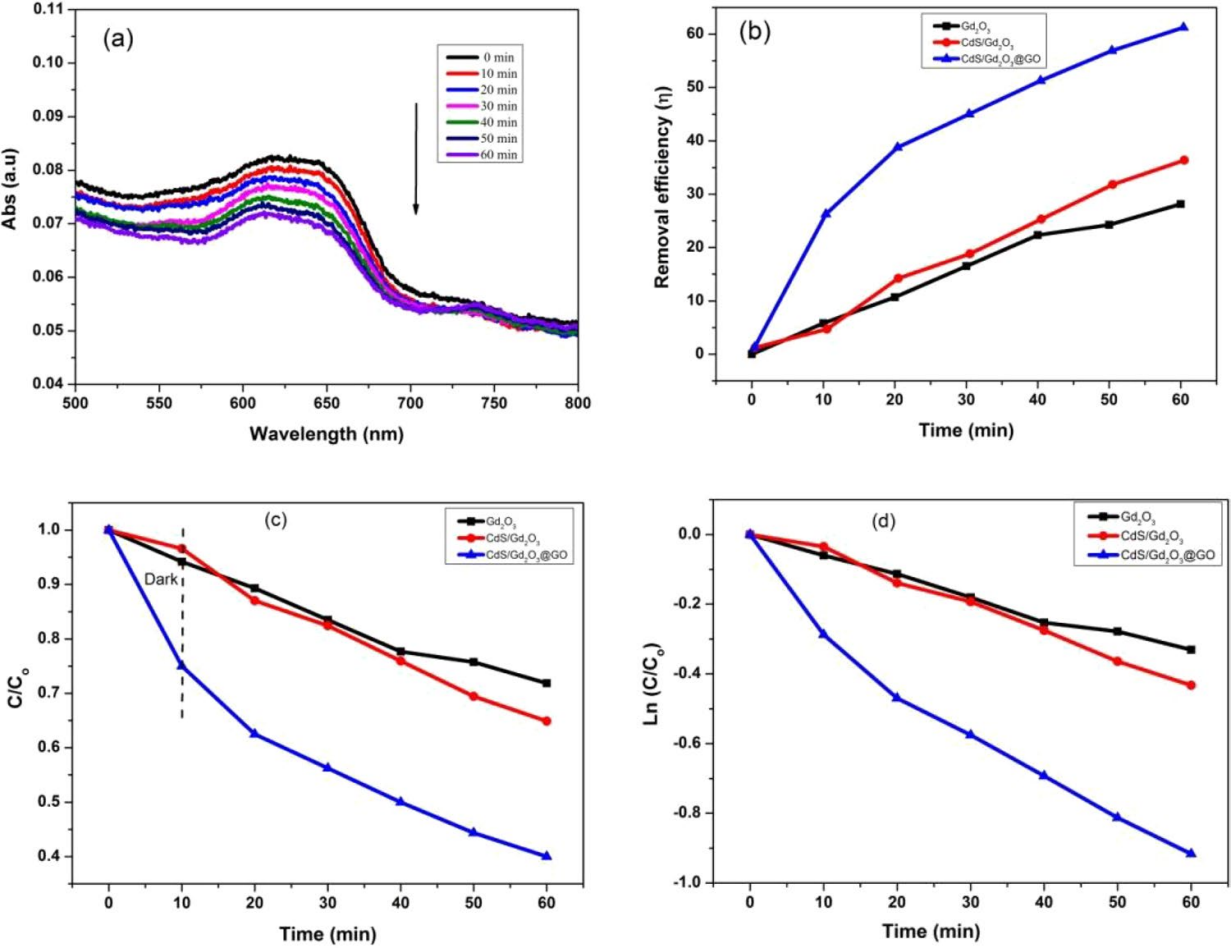
**a** Adsorption peaks of powdered CdS/Gd_2_O_3_@GO under visible light with 0.5 ppm, **b** degradation efficiency with 0.5 ppm, **c** plot *C*/*C*_0_ and time with 0.5 ppm, **d** the identical pseudo-first-order kinetics of compounds for the degradation of MB with 0.5 ppm

Figure 10a demonstrates that the reduction in the adsorption of the MB solution when the duration of the visible illumination is increased to 60 min in the presence of Gd_2_O_3_, CdS/Gd_2_O_3_, and CdS/Gd_2_O_3_@GO catalysts. The removal efficiencies of MB from the aqueous solution were analyzed by utilizing the prepared photocatalysts under visible light. The photodegradation efficiencies of Gd_2_O_3_, CdS/Gd_2_O_3_, and CdS/Gd_2_O_3_@GO were about 28.15%, 35.11%, and 60.45%, respectively. In addition, it can be obviously seen that the CdS/Gd_2_O_3_/GO composition has higher photodegradation efficiency than Gd_2_O_3_ and CdS/Gd_2_O_3_ as displayed in Fig. 10b. The removal efficiencies of Gd_2_O_3_, CdS/Gd_2_O_3_, and CdS/Gd_2_O_3_/GO for MB are exhibited in Fig. 10c. During the dark period, MB was absorbed by the surface of compounds and the adsorption equilibrium range is within 60 min. The values of the reaction rate are realized by the fitting in Fig. 10d. The reaction rate constant (*k*_app_) of the photocatalytic degradation for the Gd_2_O_3_, CdS/Gd_2_O_3_, and CdS/Gd_2_O_3_/GO were 0.0056, 0.0074, and 0.0014 min^−1^, respectively.

#### Photocatalytic activity of fiber compositions under visible and UV light with concentration 0.25 ppm

**Fig. 11:**
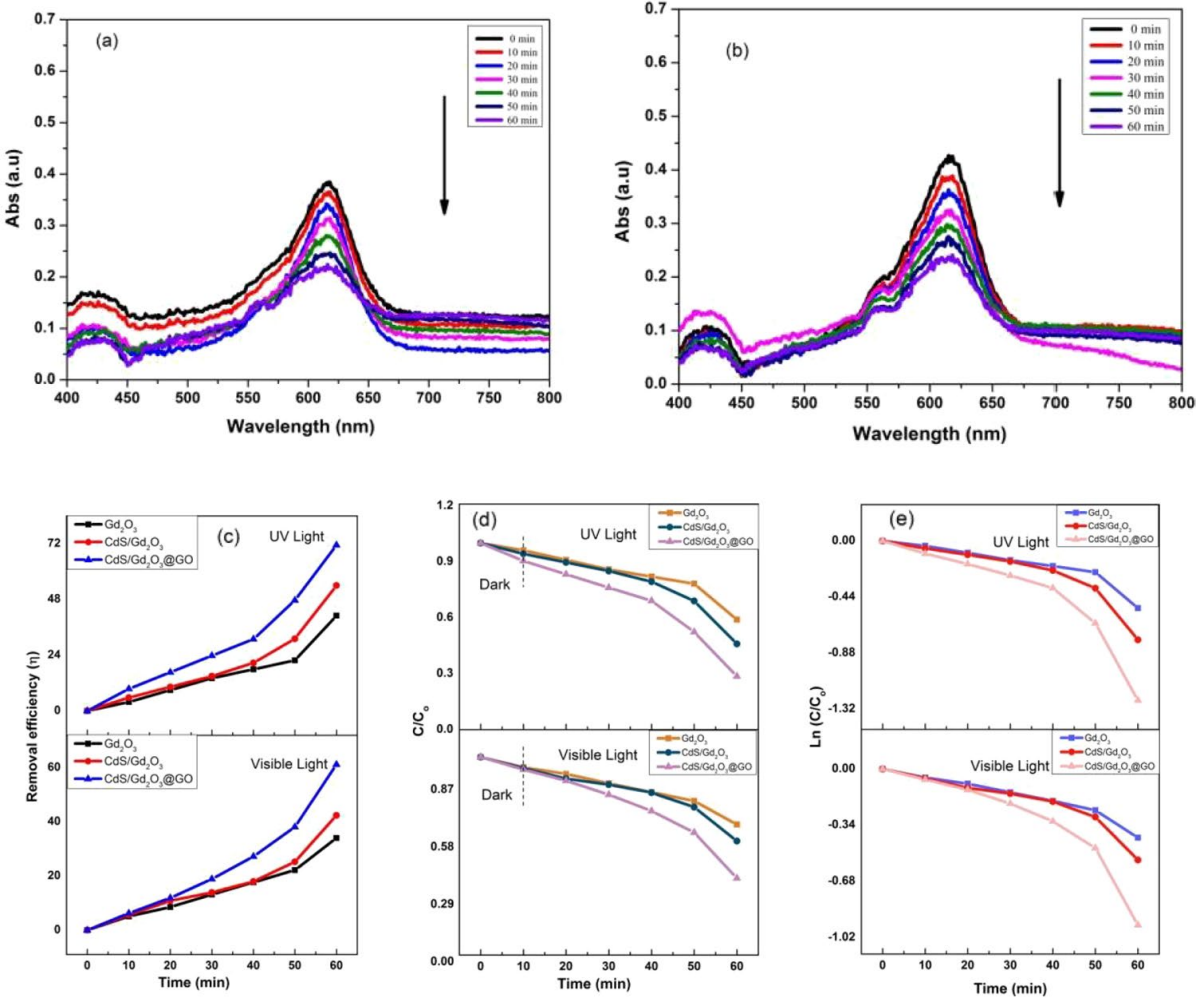
**a**, **b** Adsorption peaks of fiber CdS/Gd_2_O_3_@GO under visible and UV light with 0.25 ppm, **c** degradation efficiency with 0.25 ppm, **d** plot *C*/*C*_0_ and time with 0.25 ppm, **e** the identical pseudo-first-order kinetics of compounds for the degradation of MB with 0.25 ppm

The efficiency of nanofibers based on CA comprising the three inorganic composites degenerate MB from aqueous solutions under visible and UV light. Figure 11a and b shows the adsorbance of the MB with wavelength. The CdS/Gd_2_O_3_/GO@CA photocatalysts deteriorate more rapidly with increasing time. Figure 11c displays the MB photodegradation under visible and UV light exposure. CdS/Gd_2_O_3_/GO@CA manifests better degradation efficiency around 61.13% in comparison to Gd_2_O_3_@CA and CdS/Gd_2_O_3_@CA under visible light. In contrast, the degradation efficiency of CdS/Gd_2_O_3_/GO@CA composite (71.42%) is gradually increased as compared to that of Gd_2_O_3_@CA and CdS/Gd_2_O_3_@CA under UV irradiation. The nanofibers were left in the samples for 60 min to obtain the adsorption equilibrium state. The plot *C*/*C*_0_ varied with time as clarified in Fig. 11d. Moreover, Fig. 11e indicates the relation between ln(*C*/*C*_0_) and degradation time (*t*) under UV irradiation. The apparent rate constant (*k*1) and *R*^2^ are reported in Table 7.

#### Photocatalytic activity of fiber compositions under visible and UV light with concentration 0.5 ppm

**Fig. 12:**
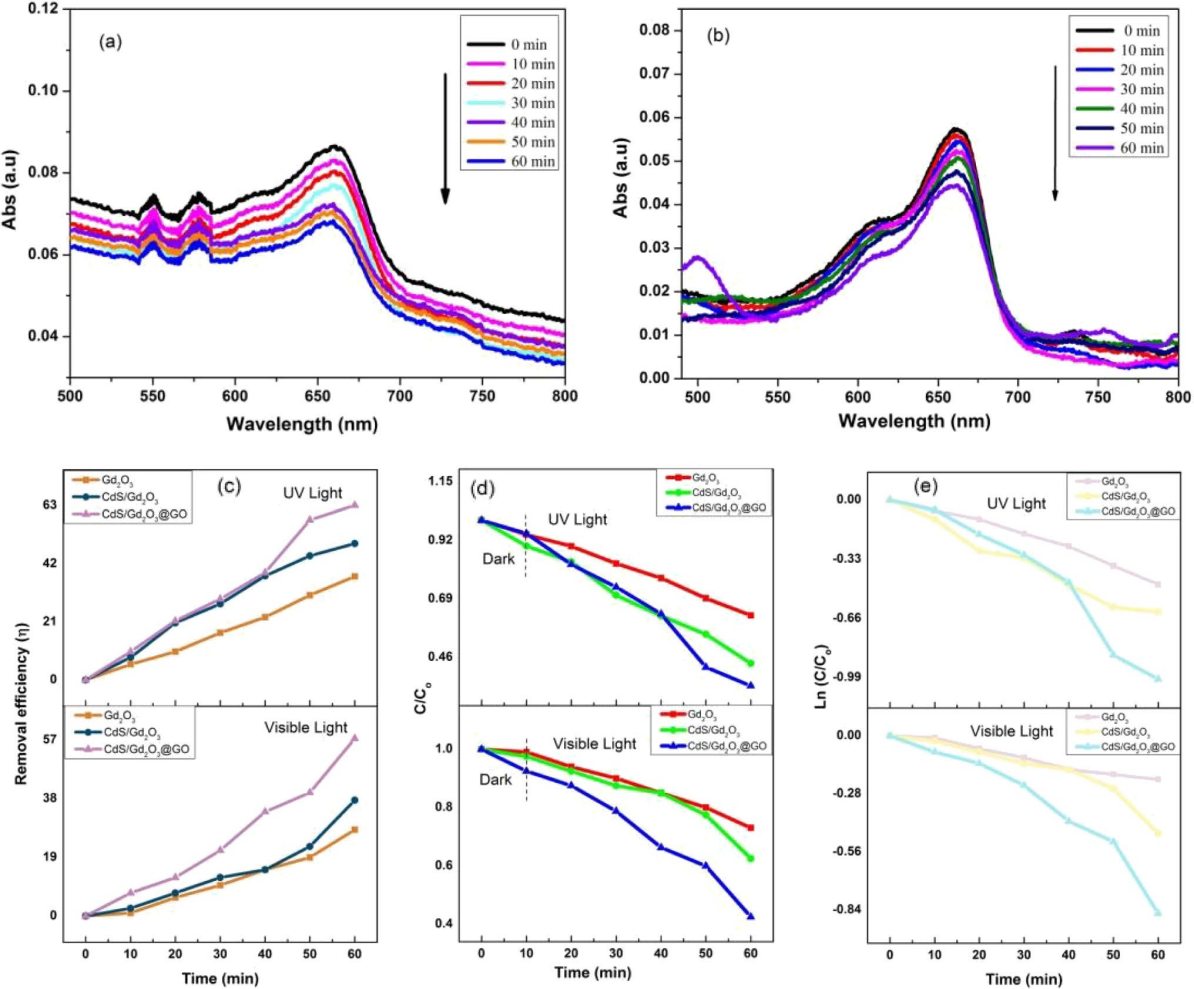
**a**, **b** Adsorption peaks of fiber CdS/Gd_2_O_3_@GO under visible and UV light with 0.5 ppm, **c** degradation efficiency with 0.5 ppm, **d** plot *C*/*C*_0_ and time with 0.5 ppm, **e** the identical pseudo-first-order kinetics of compounds for the degradation of MB with 0.5 ppm

Plot adsorbance versus wavelength as a function of time reveals a distinguished adsorbance crest at 664 nm (see Fig. 12a, b). Moreover, the removal efficiency increases from 28%, 38.75%, to 57.5% for Gd_2_O_3_@CA, CdS/Gd_2_O_3_@CA, and CdS/Gd_2_O_3_/GO@CA under visible light, respectively. Also, the efficiency (*η*) is 37.5%, 46.37%, and 63.15% for the nanofiber membranes under UV irradiation as displayed in Fig. 12c. In comparison, the plots of *C*/*C*_0_ with varied time (up to 60 min) are shown in Fig. 12d. Surveying the kinetics of degradation, the pseudo-first-order kinetic constant (*k*_app_) is matched with Ln (*C*/*C*_0_), which donates a straight line with a slope coinciding with *k*_app_ value. The values of *k*_app_, *k*1, and *q*_max_ are listed in Tables 8 and 9, while the efficiencies are given in Table 10. Table 11 displays different factors in electrospinning in the present work and previous articles.

**Table 8:** The values of *k*_app_ (min^−1^) for powder and nanofiber compounds with concentrations of 0.5 ppm are listed

Catalysts		Light source	*k*_app_ (min^−1^) × 10^−3^	*R*^2^
Powder	Gd_2_O_3_		5.6	0.989
	CdS/Gd_2_O_3_	Visible	7.4	0.991
	CdS/Gd_2_O_3_@GO	*(Halogen lamp, 500 W)*	1.4	0.954
	Gd_2_O_3_		3.8	0.974
	CdS/Gd_2_O_3_	Visible	6.9	0.846
Nanofiber	CdS/Gd_2_O_3_@GO	*(Halogen lamp, 500 W)*	1.32	0.905
	Gd_2_O_3_		7.7	0.977
	CdS/Gd_2_O_3_	UV	1.08	0.974
	CdS/Gd_2_O_3_@GO	*(UVC lamp, 15 W)*	1.7	0.924

**Table 9:** Kinetic parameters of different compositions for the Langmuir–Hinshewood (L–H) model under visible and UV irradiation with a concentration of 0.5 ppm

Catalysts		Light source	*k*1	*q*_max_
	Gd_2_O_3_		− 0.1	33.33
Powder	CdS/Gd_2_O_3_	Visible	− 0.26	12.82
	CdS/Gd_2_O_3_@GO	*(Halogen lamp, 500 W)*	− 0.1333	25
	Gd_2_O_3_		− 0.1	40
	CdS/Gd_2_O_3_	Visible	− 0.08	50
Nanofiber	CdS/Gd_2_O_3_@GO	*(Halogen lamp, 500 W)*	− 0.08	50
	Gd_2_O_3_		− 0.088	45.45
	CdS/Gd_2_O_3_	UV	− 0.0688	58.13
	CdS/Gd_2_O_3_@GO	*(UVC lamp, 15 W)*	− 0.0188	212.766

**Table 10:** The comparison between compositions, types of irradiation, and different concentrations

Composition	Catalysts	Concentration	Irradiation	Efficiency (%)
Powder	Gd_2_O_3_			42.85
	CdS/Gd_2_O_3_	0.25 ppm	Visible	60.37
	CdS/Gd_2_O_3_@GO			82.35
Powder	Gd_2_O_3_			28.15
	CdS/Gd_2_O_3_	0.5 ppm	Visible	35.11
	CdS/Gd_2_O_3_@GO			60
Powder	Gd_2_O_3_			65
	CdS/Gd_2_O_3_	0.25 ppm	UV	77
	CdS/Gd_2_O_3_@GO			91.07
Nanofiber	Gd_2_O_3_			34.03
	CdS/Gd_2_O_3_	0.25 ppm	Visible	42.37
	CdS/Gd_2_O_3_@GO			61.13
Nanofiber	Gd_2_O_3_			41.02
	CdS/Gd_2_O_3_	0.25 ppm	UV	54.71
	CdS/Gd_2_O_3_@GO			71.42
Nanofiber	Gd_2_O_3_			28
	CdS/Gd_2_O_3_	0.5 ppm	Visible	38.75
	CdS/Gd_2_O_3_@GO			57.5
Nanofiber	Gd_2_O_3_			37.5
	CdS/Gd_2_O_3_	0.5 ppm	UV	46.37
	CdS/Gd_2_O_3_@GO			63.15

**Table 11:** Different factors in electrospinning in the present work and previous articles

Composition type	Polymer concentrations (wt.%)	Voltage (kV)	Flow rate (mL/h)	Distance (cm)	Fiber morphology	Diameter (nm)	Temperature	Performance	Ref
Cellulose acetate (CA)	10	18	1	16	Cross-linked network	25.7–69.3 nm	23- 26°C	Water treatment	[present work]
PSU, DMF	20	16	2.5	10	–	–	20–25°C	Wastewater treatment	(Sakthipandi 2020; Halim et al. 2021)
Cellulose triacetate (CTA)	8	25–27	10	10	–	340–110	25°C	Desalination	(Subrahmanya et al. 2021)
PVP, absolute ethanol	10	14	1	–	–	200–600	–	Heavy metal removal	(Subrahmanya et al. 2021)
PES/HPC	10/2	7.5	1.0	–	Beads with fiber	184.1	–	Wastewater treatment	(Pervez et al. 2022)
Polyacrylonitrile (PAN) with DMF	10	20	18	0.8	Well-uniform fibers, very few beads	304–394	–	Water treatment	(Ijaz et al. 2023)

### Photocatalytic degradation mechanism

**Fig. 13:**
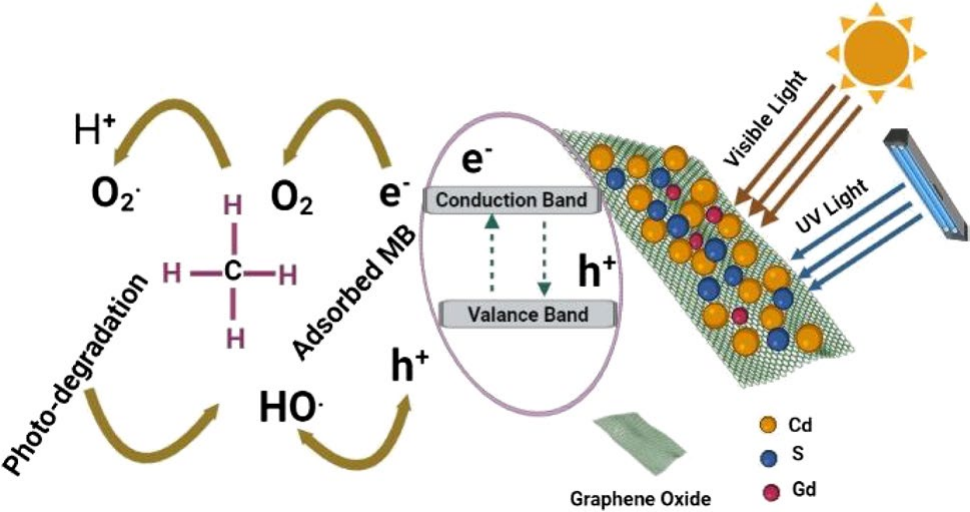
Schematic of the expected photodegradation mechanism

The MB molecules can be degenerated during both the reduction and the oxidation processes (Xing et al. 2021; Zaman et al. 2022). In the photocatalytic mechanism, the MB degradation is due to the existence of CdS, Gd_2_O_3_, and GO nanostructures under visible and UV irradiation as shown in Fig. 13. The source of energy is to improve the rate of a chemical reaction without involvement in the reaction (AlAbduljabbar et al. 2021). Under light irradiation, the electrons absorb energy in the valence band (VB) to jump to the conduction band (CB) (Liu et al. 2021), leaving a hole (h^+^) at the VB (Xue et al. 2020). Clearly, the photocatalytic activity relies on such parameters as the generation ratio of the holes and electrons, light absorption capacity, separation activity of the photogenerated holes and electrons, and photo-oxidation reduction reaction at the catalytic surface (Cruz-González et al. 2020). In addition, the photocatalytic reaction contains three main active groups h^+^, O_2_^•−^, and ^•^OH species, where the ^•^OH group is the major oxidizing agent in the degradation of organic pollutants (Yao et al. 2021). It is assumed that the electrons in CB can interact with oxygen molecules and modify oxygen radicals (O_2_^−•^) (Zhang et al. 2021). Thus, it is potential that several of the O_2_ in CB interacts with the H_2_O to compose H_2_O_2_ which absorbs light and creates hydroxyl radicals (OH^•^) (Ni et al. 2021). Despite this, the hole (h^+^) reacts with hydroxyl ion (OH^−^) and generates active OH^•^ (Prasad et al. 2020). These radicals may interact with MB dye molecules leading to degradation (Varma et al. 2020). Thus, the comprehensive degradation mechanism of MB dye under visible and UV light irradiation is dominated by OH^•^ and O_2_^−•^ radicals (Li et al. 2020c).

The itemized degradation procedure is determined using the following steps:

- Step 1: Electron–hole pair generation.
  $$CdS+h\upsilon\rightarrow electron\left(e^-\right)+hole\;(h^+)$$
- Step 2: Reduction and oxidation process.
  $${H}_{2}{O}_{2}+{O}_{2}\to {H}_{2}{O}_{2}+O{H}^{\bullet }$$
  $${H}_{2}{O}_{2}+ h\upsilon \to 2O{H}^{\bullet }$$
  $$Dye+{xO}^{\bullet }\to {CO}_{2}+{H}_{2}O$$
  $$Dye+ \dot{O H}\to\;Degradation\;Products$$
  $$Dye+h^+\left(VB\right)\rightarrow\;Oxidation\;Products$$
  $$Dye+ {e}^{-}\left(CB\right)\to\;Reduction\;Products$$

## Conclusion

The powdered compositions Gd_2_O_3_, CdS/Gd_2_O_3_, and CdS/Gd_2_O_3_@GO were prepared. The crystallite size “*D*_s_” of the prepared nanocomposites Gd_2_O_3_, CdS/Gd_2_O_3_, and CdS/Gd_2_O_3_@GO was estimated to be 29.62, 11.62, and 11.56 nm, respectively. Photocatalytic activity of MB degradation was investigated within 60 min in both visible and UV light as follows. In case of concentration 0.25 ppm MB, the degradation efficiency (*η*) reaches the highest values at 42.85%, 60.37%, and 82.35% in visible light, while it extends the values to 65%, 77%, and 91.07% for Gd_2_O_3_, CdS/Gd_2_O_3_, and CdS/Gd_2_O_3_@GO under UV light. On the other hand, in case of concentration 0.5 ppm, *η* reaches the values of 28.15%, 35.11%, and 60% for Gd_2_O_3_, CdS/Gd_2_O_3_, and CdS/Gd_2_O_3_@GO under visible light. Regarding the nanofiber compositions with a concentration 0.25 ppm, the photocatalytic features of the Gd_2_O_3_@CA, CdS/Gd_2_O_3_@CA, and CdS/Gd_2_O_3_/GO@CA are characterized under visible and UV light irradiation. The highest photodegradation activity (71.42%) was found for the nanofiber composite CdS/Gd_2_O_3_/GO@CA under UV irradiation, in comparison to an activity (61.13%) under visible light. This nanofiber composite could be employed in abundant applications and particularly for the degradation of organic pollutants.

## Data Availability

Data will be made available on request.
